# Insights from 20 years of bacterial genome sequencing

**DOI:** 10.1007/s10142-015-0433-4

**Published:** 2015-02-27

**Authors:** Miriam Land, Loren Hauser, Se-Ran Jun, Intawat Nookaew, Michael R. Leuze, Tae-Hyuk Ahn, Tatiana Karpinets, Ole Lund, Guruprased Kora, Trudy Wassenaar, Suresh Poudel, David W. Ussery

**Affiliations:** 1 Comparative Genomics Group, Biosciences Division, Oak Ridge National Laboratory, Oak Ridge, TN 37831 USA; 2 Joint Institute for Biological Sciences, University of Tennessee, Knoxville, TN 37996 USA; 3 Department of Microbiology, University of Tennessee, Knoxville, TN 37996 USA; 4 Computer Science and Mathematics Division, Computer Science Research Group, Oak Ridge National Laboratory, Oak Ridge, TN 37831 USA; 5 Center for Biological Sequence Analysis, Department of Systems Biology, The Technical University of Denmark, Kgs. Lyngby, 2800 Denmark; 6 Molecular Microbiology and Genomics Consultants, Tannenstr 7, 55576 Zotzenheim, Germany; 7 Genome Science and Technology, University of Tennessee, Knoxville, TN 37996 USA

**Keywords:** Bacteria, Comparative genomics, Bacterial genomes, Metagenomics, Core-genome, Pan-genome, Next-generation sequencing

## Abstract

Since the first two complete bacterial genome sequences were published in 1995, the science of bacteria has dramatically changed. Using third-generation DNA sequencing, it is possible to completely sequence a bacterial genome in a few hours and identify some types of methylation sites along the genome as well. Sequencing of bacterial genome sequences is now a standard procedure, and the information from tens of thousands of bacterial genomes has had a major impact on our views of the bacterial world. In this review, we explore a series of questions to highlight some insights that comparative genomics has produced. To date, there are genome sequences available from 50 different bacterial phyla and 11 different archaeal phyla. However, the distribution is quite skewed towards a few phyla that contain model organisms. But the breadth is continuing to improve, with projects dedicated to filling in less characterized taxonomic groups. The clustered regularly interspaced short palindromic repeats (CRISPR)-Cas system provides bacteria with immunity against viruses, which outnumber bacteria by tenfold. How fast can we go? Second-generation sequencing has produced a large number of draft genomes (close to 90 % of bacterial genomes in GenBank are currently not complete); third-generation sequencing can potentially produce a finished genome in a few hours, and at the same time provide methlylation sites along the entire chromosome. The diversity of bacterial communities is extensive as is evident from the genome sequences available from 50 different bacterial phyla and 11 different archaeal phyla. Genome sequencing can help in classifying an organism, and in the case where multiple genomes of the same species are available, it is possible to calculate the pan- and core genomes; comparison of more than 2000 *Escherichia coli* genomes finds an *E. coli* core genome of about 3100 gene families and a total of about 89,000 different gene families. Why do we care about bacterial genome sequencing? There are many practical applications, such as genome-scale metabolic modeling, biosurveillance, bioforensics, and infectious disease epidemiology. In the near future, high-throughput sequencing of patient metagenomic samples could revolutionize medicine in terms of speed and accuracy of finding pathogens and knowing how to treat them.

## Introduction

Two decades have passed since the first bacterial genome was completely sequenced (Fleischmann et al. 1995; Fraser et al. 1995), and the technical improvements and subsequent increases in biological knowledge have been just as dramatic in the second 10 years as they were in the first decade. The most significant factor influencing scientific progress was, as predicted, the vast reduction in the price of sequencing, as a result of technical developments. Along with the cost reduction, second-generation (or “next-gen”) sequencing techniques dramatically reduced the average read length; in contrast, third-generation (single molecule) sequencing allows for longer read lengths, although at the time of writing, these methods are still in their infancy. The dramatic reduction in the cost of sequencing has made bacterial genome sequencing affordable to a great number of labs, leading to a democratization of sequencing (Shendure and Ji 2008). The explosive growth of data has resulted in a cost shift from sequencing to assembly, analysis, and managing data.

**Fig. 1:**
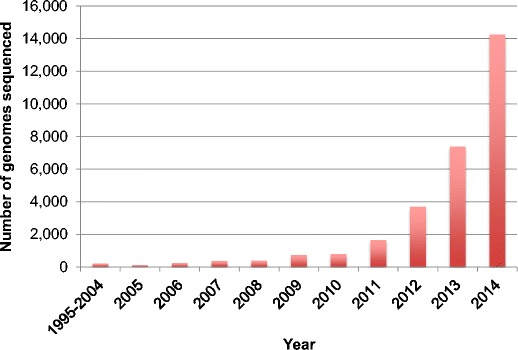
Number of bacterial and archaeal genomes sequenced each year and submitted to NCBI. Source: GenBank prokaryotes.txt file downloaded 4 February 2015

Ten years ago, we reviewed the first decade of bacterial genome sequencing (Binnewies et al. 2006). At that time, there were about 300 sequenced bacterial genomes and only two published metagenomic projects; this represented a growth of more than 100-fold from the mere two genomes sequenced in 1995. The number of sequenced genomes has continued to increase dramatically in the last 10 years (Fig. 1), growing another hundredfold—that is, there are more than 30,000 sequenced bacterial genomes currently publically available in 2014 (NCBI 2014) and thousands of metagenome projects (GOLD 2014). Projects such as the Genomic Encyclopedia of Bacteria and Archaea (GEBA) (Kyrpides et al. 2014) promise to not only add more genomes but expand the genetic diversity and add to the list of available types of strains.

For many years, ribosomal RNA (rRNA) operons, specifically the 16S rRNA genes, were used as the primary tool for taxonomic assignment and phylogenetic trees (Mizrahi-Man et al. 2013). The 16S rRNA gene is still widely used because it is present in at least one copy in every bacterial genome, its conserved regions enable simple sample identification using PCR, and its sequence provides reliable information on bacterial family, genus, or species in most cases. This single gene comparison is now being replaced by more comprehensive approaches. Full genome sequencing along with additional tools can comprehensively analyze and classify hundreds or thousands of genomes. These new tools have led to new understandings of genetic relationships that the 16S rRNA gene only approximates.

A notable development in the second decade of bacterial genome sequencing was the generation of metagenomic data, which covers all DNA present in a given sample (Mende et al. 2012). The study of metagenomes was so new in the last review that the term needed to be defined, as at that time there were only two metagenomic projects published. Today, there are more than 20,000 metagenomic projects publically available, and many terabytes of sequencing data have been produced. The myriad of ecosystems includes numerous animal and human microbiomes, soils of all types, fresh and salt water samples, and even plant–microbe interaction systems.

As observed 10 years ago, the diversity of bacteria continues to expand and surprise (Lagesen et al. 2010). Instead of 20 *Escherichia coli* genomes, we now have thousands that can be compared (Cook and Ussery 2013), and they still give us new insights into the diversity and plasticity of bacterial genomes.

The nature of data to be analyzed is changing. For example, microarray analysis of transcriptomes is being replaced by RNA sequencing (Wang et al. 2014; Westermann et al. 2012; Zhao et al. 2014), which has some substantial advantages, although the statistical analysis packages for this data are continually evolving and are by no means standardized. The stories revealed from analysis of these sample metagenomes, especially the human microbiomes, have dramatically changed our view of the microbial world to the point that the general public is now aware of the possible beneficial effects of bacteria on their health and not just as the source of illness (Claesson et al. 2012; Huttenhower et al. 2012).

The ever-increasing amount and complexity of generated sequences has large implications for analysis of this data. The bioinformaticists’ ability to analyze, compare, interpret, and visualize the vast increase in bacterial genomes, transcriptomes, proteomes, metatranscriptomes, etc., is valiantly trying to keep up with these developments. Most biologists are drowning in too much data, and in desperate need for tools to help them make sense of their massive amounts of sequences. It seems clear that these trends will continue for the foreseeable future as genome data becomes cheap and abundant. As will be discussed later, there are many new methods available for help with this, but it is likely that there will be a continued demand for good bioinformatics tools.

There are numerous new assembly algorithms being developed to deal with the output of new sequencing technologies, and these will have to continue to evolve as the third-generation sequencing technology comes online (El-Metwally et al. 2013). Traditional genome annotation pipelines are no longer able to scale to the rate of sequence production and new approaches are continually being considered (Nielsen et al. 2014; Pop 2009). The number of published genomes will no longer allow “all vs. all” comparisons without access to large computer clusters or “supercomputers,” unless new and more computationally efficient algorithms are developed and new ways to visualize and communicate the results.

Here, we present some insights that have emerged from numerous bacterial sequencing projects. We are unable to cite all important and influential papers that have contributed to these insights, in addition to the many genome sequences that have been submitted to public databases. We wish to express our gratitude to all colleagues who have shared their data with the scientific community, without which far less scientific progress would have been possible.

## Overview of available data

**Fig. 2:**
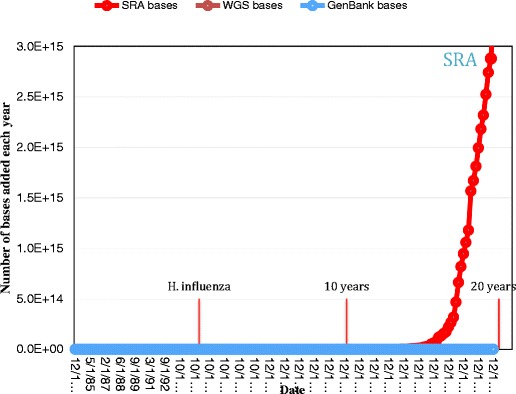
Number of bases added each year since 1982. The dates for the first bacterial genome (*H. influenzae*) to be sequenced, and 10- and 20-year anniversaries are marked. Due to the scale, WGS and GenBank bases are essentially flat. Source: GenBank and SRA, accessed 4 February 2015

In 1995, when the first bacterial genomes were sequenced, GenBank had already grown more than 500-fold from when it was first started, in 1982. Ten years later, as automated sequencing became more common, GenBank had grown to more than 75,000 times its original size. Almost 20 years later, at the time of writing this article, complete genomes in GenBank appear to be slowing down a bit in favor of other types of submissions. Starting with their introduction in 2002, WGS bases have kept pace with or exceeded GenBank bases and the addition of Sequence Read Archive (SRA) bases in 2008 have dwarfed them both (Fig. 2).

As of January 2015, the SRA contained more than 1500 trillion (10^15^) nucleotides or 8000 times the size of GenBank and Ensemble (Ensemble 2015) had over 20,000 single isolate genomes. Indicators of the genomes in process include the Genomes Online Database (GOLD) (Pagani et al. 2012), which had 47,083 prokaryotic genomes and the MG-RAST system listed 152,927 metagenomes, of which 23,242 are publically accessible. There is no reason to believe that this trend will stop any time soon or that the insights found will be any less profound.

The Ensemble genomes are from 61 phyla, 1600 genera, and 9800 species. The six phyla *Actinobacteria*, *Bacteroidetes*, *Cyanobacteria*, *Firmicutes*, *Proteobacteria*, and S*pirochaetes* together represent 96 % of the data. No other phyla represents over 1 % of total genomes (Table 1). Note that currently almost half of all the genomes sequenced are from the Proteobacteria phylum. In the future, as unculturable genomes from metagenomic samples and third-generation sequencing continue to make their way into the international databases, the distribution of the phyla and number of species will likely change.

**Table 1:** Number of sequenced genomes for 6 selected phyla and the percent of all genomes found in the phyla

Phyla	Number genomes	% of total
Actinobacteria	4059	13
Bacteroidetes/Chlorobi group	932	3
Cyanobacteria	340	1
Firmicutes	9628	31
Proteobacteria	14,268	46
Spirochaetes	525	2
Other	1500	5

Source: GenBank prokaryotes.txt file downloaded 4 February 2015

### Annotation and deciphering of the genomes

For the finished genomes, a few broad conclusions can be made. First, the average protein coding content of a bacterial genome is 88 % for the 2671 finished genomes in GenBank; however, the range is from just under 40 to 97 % (Land et al. 2014). Although a “typical” bacterial genome is around 5 million bp and encodes about 5000 proteins, the range of sizes is quite broad—more than a hundredfold. The largest genome currently (January 2014) that is complete and in GenBank is *Sorangium cellulosum* strain So0157-2, at 14,782,125 bp, and contains 11,599 genes (Han et al. 2013). The smallest bacterial genome sequenced is Candidatus *Nasuia deltocephalinicola* strain NAS-ALF; the genome encodes a mere 137 proteins, and is only 112,091 bp in length (Bennett and Moran 2013).

**Fig. 3:**
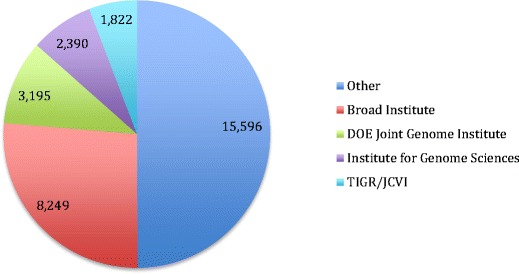
Number of genome sequences from the largest four sources. All sources with less than 1000 genomes are combined in the “Other” category. Source: GenBank prokaryotes.txt file downloaded 4 February 2015

As the number of sequenced organisms expands, no one person can have a working acquaintance with every sequenced genus. As a result, the quality and richness of the metadata take on greater importance. Many sequenced samples were never characterized phenotypically, physiologically, or metabolically and the sampling details may be buried in the literature. To address this need, standards have been developed for the minimum metadata that should be included with sequence data (Kottmann et al. 2008). Metadata is usually more available for recent genomes and more for finished over permanent draft genomes. We have analyzed the metadata of genomes publically available in the Integrated Microbial Genomes comparative analysis system (IMG) (Markowitz et al. 2014) and GOLD (Pagani et al. 2012). The genomes come from a diverse group of institutions with nearly half of the genomes coming from three sequencing centers (Fig. 3).

The GOLD database has collected ecosystem information on roughly 20,000 of its 59,000 samples. This field is now mandatory in GOLD submissions and it provides a profile of recently registered projects. About 58 % of the declared ecosystems are from host-associated environments, and of those, the largest group is human-associated genomes (Table 2).

**Table 2:** Number of genomes found within each GOLD-defined ecosystem

Ecosystem	Total
Host-associated	11,816
Humans	4973
Animal	1804
Plants	1410
Mammals	867
Other	2762
Environmental	6774
Aquatic	4559
Terrestrial	2057
Other	158
Engineered systems	1658
Food production	440
Wastewater	410
Lab synthesis	387
Other	418
Total	20,248

Source: GOLD, accessed 4 February 2015

**Fig. 4:**
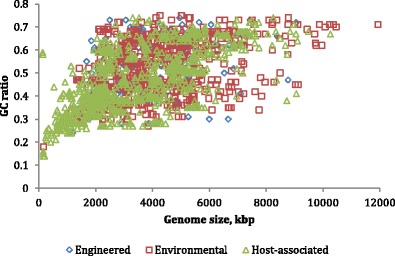
Genome size and percent GC of 2139 finished genomes plotted for the ecosystem types of (1) engineered systems, (2) environmental sources, and (3) host-associated genomes. Source: GOLD, accessed 4 February 2015

In agreement with previous observations from analyses of a smaller set of organisms (Bentley and Parkhill 2004; Bohlin et al. 2010; Karpinets et al. 2012), genomes of bacteria from complex environmental habitats have a tendency to be larger in size and have greater GC content than those of the host-associated bacteria (Fig. 4). The GC content of the finished bacterial genomes ranges from a bit less than 15 % to about 85 %.

Although many bacteria are mesophiles, there are a growing number of sequenced extremophiles, such as thermotolerant, psychrotolerant, and psychrotrophic bacteria (Table 3) (IMG 2014).

**Table 3:** Number of genomes found within each temperature range

Temperature range	Number genomes
Mesophile	3173
Thermophile	171
Hyperthermophile	75
Psychrophile	36
Psychrotolerant	17
Psychrotrophic	6
Thermotolerant	3
Unknown	20,626

Source: IMG Metadata Categories, accessed 4 February 2015

### Three generations of sequencing

Ten years ago, most genomes were still sequenced by the Sanger method, which was mainly performed using a factory production model with robots selecting and growing clones of whole genome shotgun libraries, isolating sequencing templates, and performing the sequencing reactions, followed by electrophoresis on a bank of 96 or 384 well capillary machines. The output from such production lines was then automatically assembled and usually generated a high-quality draft of the genome. Finishing these draft genomes was much more labor intensive and required a separate production line to be efficient. The cost of a finished bacterial genome could amount to as much as $50,000 and was approximately equally divided between creating the draft genome and finishing it. Despite these considerable costs, most bacterial genomes were finished to completion when made public. Due to the technical requirements, the vast majority of bacterial genome sequencing projects were restricted to a few large sequencing centers.

The development of low-cost and reasonably high-throughput “Next-Generation Sequencing” (NGS) opened a market for commercial vendors. The cost of producing raw sequence data declined to the point that it currently can cost less than $1 to generate a draft bacterial genome. This in turn, has made sequencing bacterial genomes both cost effective and obligatory for almost any research team.

These newer, second-generation sequencing technologies (initially introduced by Roche 454, now Illumina is commonly used) produced considerably shorter reads than Sanger sequencing. One consequence was an increase in the recommended coverage needed for an assembly and a larger number of contigs that needed closure before a genome was finished to completion. While the cost of producing a draft genome was significantly reduced, the cost ratio between a draft and a complete sequence was dramatically changed. The costs of finishing a genome could amount to over 95 % of the total, and therefore arguably was no longer cost effective.

As a result, more and more genomes were published while still in multiple contigs and varying quality. A set of standards for the quality of submitted genomes has been published previously (Chain et al. 2009). The fraction of draft genomes has grown dramatically, with debate over the relative value and cost-effectiveness of finishing bacterial genomes to completion. Recently, we estimated genome quality scores for more than 32,000 genomes and found that for most purposes, most genomes are “good-enough” quality, with only about 10 % of the draft genomes being of too-poor quality to use (Land et al. 2014). Mavromatis et al. compared draft versions of genomes to their finished version and concluded that Illumina-based sequencing was a cost-effective approach for generating draft microbial genomes without a significant loss of information (Mavromatis et al. 2012). Even though the process of finishing genomes is currently still time-consuming and costly, it will continue to have a role in establishing reference genomes that are used in assembly of other members of a species.

As of January 2015, a large percentage of bacterial genomes are still in draft status (Table 4) and have an average of 190 contigs compared with an average of 5 contigs for genomes defined as “finished” (Land et al. 2014). This increased number of contigs creates a major challenge for comparative analysis and raises questions about the accuracy of the basic genomic characteristics of draft genomes, such as the genome size, the number of predicted genes, number of repeats, and the GC content.

**Table 4:** Number of complete and permanent draft genomes and the percent of those genomes with each project status

Project status	Bacteria	Archaea	Plasmids	Total
Finished	3060	173	1186	4419
Permanent draft	19,696	312	9	20,017
Draft	672	4	1	677
Total	23,428	489	1196	25,113

Source: IMG Statistics, accessed 4 February 2015

Third-generation sequencing (single-molecule sequencing) such as, PacBio (Brown et al. 2014; Terabayashi et al. 2014) and MinION (Mikheyev and Tin 2014; Quick et al. 2014), can produce much longer reads (several thousand bp) compared with the NGS technology (a few hundred bp). These newer technologies hold the promise of not only generating more sequence for less money, but they may eventually eliminate the concept of draft microbial genomes all together.

## Insights into novel genome features

### Genome size variation, protein-coding content

In contrast to the prevailing view among many bacteriologists, members of a species are not necessarily “equal” or even similar, in terms of their (protein-coding) gene content, as can be seen by the 2000 *E. coli* genomes shown in Fig. 6. Depending on the species, the variation in gene content and genome size can be quite considerable, with some pan-genomes, like *E. coli*, being very “open”; other pan-genomes, such as that for *Bacillus anthracis*, contain very few extra genes, and can be considered “closed”, although with viruses and other mobile elements, there can always be a few new genes. Thus in our opinion, there is no such thing as a closed pan-genome, but merely a “less open” one. Whereas some species comprise a very confined and homogeneous group of strains, in which genetic variation is mostly seen in mobile DNA elements and single nucleotide polymorphisms (SNPs) (e.g., *Mycobacterium tuberculosis*), there are other species containing members whose genome size varies considerably. It could be argued that the observed size range in *E. coli* is due to the large number of available sequenced strains; however, even less frequently sequenced species can vary by more than a megabase (*Haemophilus influenzae* HK1212 (1.0 mb) versus F3047 (2.0 mb) and *Burkholderia pseudomallei* THE (6.3 mb) versus MSHR520 (7.6 mb)).

The average protein-coding density of bacterial genomes is 87 % with a typical range of 85–90 % (McCutcheon and Moran 2012). As mentioned above, protein-coding density for some genomes can be less than 40 %. Many of these are symbionts, obligate pathogens, or have a large number of pseudogenes. For example, *Serratia symbiotica* str. *Cinara cedri* has a protein-coding density of 38 %, is an insect co-symbiont, has been and is still going through a substantial genome reduction, and contains at least 58 pseudogenes (Lamelas et al. 2011). *Nostoc azollae 0708* is a symbiont of a fresh water fern, and although it appears to have a much higher coding density at 52 %, it is lower than any other cyanobacteria. Related to other free-living *Nostocs* and *Anabeanas*, it is no longer capable of independent living, is undergoing active genome decay, and about 30 % of its identifiable coding regions are pseudogenes (Ran et al. 2010). In contrast, the cyanobacteria *Trichodesmium erythraeum* IMS101 with a gene density of 63 % also contains a large number of pseudogenes (12 %) but without obvious environmental pressures (Pfreundt et al. 2014). It is a free living, filamentous, colony-forming, nitrogen-fixing, bloom-causing cyanobacteria that lives in tropical and subtropical oceans none of which fit the known reasons for going through a genome reduction.

Many genomes contain a lot of redundancy, in terms of gene duplications, as well as pseudogenes that seem to have lost any function. Together with repeat sequences and parasitic DNA that seem to bear no function to the organism, the only conclusion can be that bacterial genomes are not always evolving towards optimal efficiency. The presence of such “junk” DNA is one reason for the vast variation in genome size within the bacterial world, although the genome’s size is of course also dependent on the number of functional genes and pathways that are present. The latter roughly correlates to the diversity of growth conditions an organism can endure.

### Genetic diversity is much greater than we thought

In our review of the first decade of bacterial genomics, we concluded that the genomic diversity of the bacterial world is far greater than expected (Binnewies et al. 2006). Even within a species, there can be a large degree of genetic variation. This conclusion is still valid and has now been shown to exist across most of the bacterial and archaeal world. Our initial conclusion was primarily based on a comparison of 20 *E. coli* genomes and stated that any one of these genomes would have at least 100 genes unique to that strain. Obviously, now 10 years later, with genomes from 50 different phyla, many things are quite different from *E. coli*, with some genomes containing only a tiny handful of genes in common with *E. coli*. Furthermore, even within *E. coli*, there is stunning diversity, as can be seen in Fig. 6; any one *E. coli* genome contains about 5000 genes, and roughly two-thirds of these are found in all *E. coli* genomes, but the other third are “accessory genes,” found in other strains, but not all. Surprisingly, any one *E. coli* contains less than 10 % of the total number of *E. coli* genes in the *E. coli* pan-genome. Even at the level of transcription factors, there is an enormous diversity with *E. coli* genomes (Cook and Ussery 2013). With over 2800 sequenced *E. coli* genomes now available in GenBank, it is obvious that genome comparison using sequence alignments soon will become impractical.

### Diversity in what all bacteria need: tRNAs, codons, and codon usage

All bacterial genomes have at least one copy of the 23S, 16S and 5S rRNA genes. In the vast majority of genomes these exist as an operon with a conserved structure of the 23S gene, followed by one or more transfer RNAs (tRNAs), then the 16S, the 5S, and optionally one or more additional tRNAs. There are, however, exceptions and rearrangements (Lim et al. 2012) such as *Burkholderia mallei* SAVP1 that contains two extra 16S rRNA genes by themselves and *Haloarcula marismortui* which has 5 % diversity among its 16S rRNA genes (Pei et al. 2010). The number of copies of the rRNA cistron varies from 1 to 15 (Land et al. 2014) and seems to be related to the minimum replication time for that genome (Klappenbach et al. 2000), although there seems to be some anomalous *E. coli* genomes in this regard.

The number of tRNA genes is also variable in the bacterial world. The genetic code allows for 62 possible anticodons for tRNAs, but since these have to cover only 20 essential amino acids, the theoretical minimum for a genome would be 20 tRNA genes. In reality, the number of tRNA genes and anticodons used in a genome varies but rarely approaches either of these extremes. The number of tRNA genes per genome varies from an unknown low (due to the variable quality of even some finished genomes, but presumably at least 20) to a high of 284, with an average of about 55. The number of anticodons identified per genome has not exceeded 47 (out of 62 possible) (Land et al. 2014) and averages between 33 and 35, so it seems that many anticodons are associated with multiple tRNAs, often due to base wobble in the third position. This is an example of genetic indulgence, with far more tRNA genes than codons used, in contrast to the classical view of bacterial genomes being “lean”. Other observations also point to the fact that bacteria are not always concerned with genetic efficiency. In addition, there is evidence that an increased number of tRNAs and rRNAs is correlated with a faster growth rate (Lee et al. 2009).

### Important roles for DNA sequence repeats in bacterial genomes

DNA sequence diversity among bacterial genomes from the same species is far greater than we thought. Bacteria are constantly fighting viruses, and two bacterial genomes that are closely related can contain many insertions and deletions from recombination events. A recent review of repeats affecting genomic stability has surveyed various types of mobile elements, and how bacteria can control them (through post-segregation killing systems) (Darmon and Leach 2014). Whole-genome Shotgun (WGS) sequencing has opened the doors for an expansion in the number and diversity of repeats with a defined function. There are genomes with evidence of over 1600 palindromic repeats and ones with thousands of Miniature Inverted-repeat Transposable Elements (MITEs) (Delihas 2011; Rocco et al. 2010).

The mapped diversity of transposable elements (TEs) has grown in unprecedented ways (Guerillot et al. 2014). TEs have been shown to range from about 1 to 52 kb in size and work with several families of insertion sequences (IS) and integrative and conjugative elements. The deluge of sequencing data has led to a dramatic increase in the number of identified prokaryotic transposons. The types, nature, and mechanisms of IS and transposons have received enough attention that a database for the registration and consistent nomenclature of IS elements was developed (Siguier et al. 2006).

MITEs are usually less than 300 bp, nonautonomous and do not transpose by themselves because they lack the transposase gene. They appear to be the remnant of insertion sequences, with the terminal inverted-repeat (TIR) sequence, the direct repeats and target site duplication (Delihas 2011). While they have been known for some time, the numbers, types, and genetic diversity has been greatly expanded due to the availability of genomic sequences and improved search algorithms. They are found in a broad range of organisms and RNA transcripts have been detected.

A family of uniformly spaced repeats called clustered regularly interspaced short palindromic repeats (CRISPRs) has been recognized for some time (Mojica et al. 2000). Their distribution and significance as a defense mechanism has been more fully appreciated in the last 10 years. In addition to the repeats, a family of CRISPR-associated proteins (CRISPR-Cas) is used to organize the CRISPRs in the major types and several subtypes (Makarova et al. 2011a, b; Sorek et al. 2008). The CRISPR-Cas system is thought to be a general stress response, including responses that provide a type of immunity and those that are pathogenic to the host (Louwen et al. 2014). Approximately 80 % of archaea and 40 % of bacteria have a CRISPR-Cas system that both allows them to fend off viral attacks (Grissa et al. 2007; Horvath and Barrangou 2010) and can play a role in evasion of a host’s immune system (Louwen et al. 2014). As the role in virulence is elucidated, the Cas9 protein of the system is showing promise as tool for genetically engineering new weapons in the war against antibiotic-resistant bacteria (Birkard et al. 2014).

### Defense systems in archaea and bacteria

The dramatic increase in available bacterial sequences has facilitated and accelerated a wide range of comparative analyses including the discovery that prokaryotic organisms can have up to 10 % of their genome dedicated to the defense systems (Makarova et al. 2013). Archaea and thermophiles tend to have the largest proportion of their genomes dedicated to defense and these defense genes are often localized in they tend to form genomic islands, which contain many hypothetical genes of defense genes that are larger than typical operons and transposases for horizontal transfer. Horizontal gene transfer plays an important role in the maintenance and evolution of these defense islands which have on average 5.7 genes (Makarova et al. 2011a, b).

A recent review of bacterial defense systems (Makarova et al. 2013) showed the explosive growth of genomic sequencing and analysis. This has led to a greatly expanded knowledge of these defense systems, including the discovery of novel restriction-modification systems, new toxin/anti-toxin systems, and the CRISPR-Cas immunity system. The systems have been grouped into analogs of innate immunity and adaptive immunity and infection-induced dormancy or programmed cell death. The innate immunity is based on recognition of non-self DNA and includes restriction-modification systems and DNA phosphorathioation systems. These systems modify “self” DNA in order to target non-self and fight infection without specificity. CRISPR repeats are classified as adaptive immunity because they have a memory of previous viral attacks. The dormancy and programmed cell death group includes toxin-antigen systems and abortive infection, both of which are induced by infection.

### Bacterial microcompartment organelles

A review of bacterial microcompartments (BMCs) (Chowdhury et al. 2014) describes bacterial protein structures that are organelle like and can be used to optimize metabolic pathways. They are strictly proteins with no evidence of lipid content or similarity to viral capsids and can contain up to 20,000 polypeptides. Genomic sequencing has revealed eight types of BMCs and has suggested that they are not only involved in carbon fixation but also in the metabolism of ethanol, fucose, rhamnose, and an unspecified amino alcohol (Jorda et al. 2013). They are distributed across many phyla and have been found in up to 17 % of bacteria (Jorda et al. 2013). Because BMC genes tend to be clustered with genes related to their function, available genomic sequences have led to hypotheses about the functions of nearby genes and eventually to new discoveries. There is some evidence that many BMCs be may associated with frequent HGT (Abdul-Rahman et al. 2013).

### Genome comparisons and phylogeny

A bacterial species was originally defined using a combination of morphology and simple biochemical tests such as the utilization of specific carbon, nitrogen sources, and their reaction to the Gram stain. Subsequently, the DNA-DNA hybridization (DDH) as the “gold standard” backed up bacterial species determination for more than 50 years (McCarthy and Bolton 1963; Schildkraut et al. 1961) where a DDH value of 70 % was widely accepted as the cutoff for separating bacterial species (Wayne et al. 1987). With the emergence of rapid DNA sequencing technology, the comparative 16S rRNA analysis replaced the time-consuming and labor-intensive DDH technique where a 97 % sequence identity of the full-length 16S rRNA gene was used to define a new species, with acknowledged exceptions (Goebel and Stackebrandt 1994; Stachebrandt and Ebers 2006).

Over 30 years, the 16S rRNA sequence was used for prokaryotic phylogeny inference and taxonomic classification and for inferring the microbial diversity of environmental samples. It is well known, however, that very similar 16S rRNA gene sequences can lead to poor resolution at the species level (Case et al. 2007). Instead of focusing on the one 16S rRNA gene, it is now possible to do phylogenetic profiling with genome scale analysis using reference genomes, groups of conserved proteins, or complete genomes or proteomes.

The more genes considered, the better taxonomic resolution and the less sensitivity to horizontal gene transfer (Oren and Papke 2010). A paradigm shift has taken place, from one gene-based modeling into genome-scale modeling. These genome-scale comparisons make it possible to not only improve phylogeny inference but to have better accuracy on inferring functional pathways.

These genome-scale comparisons are becoming routine and the approaches can be divided into two main categories, alignment-based (for example, an alignment of about 400 universal proteins identified by Segata et al. 2013) and alignment-free (for example, the “google DNA” method described recently; Gautier and Lund 2013). Many of the “alignment-free” methods work well for retrieving sequences already in the database but do not work as well for assigning the relative distance for distantly related genomes. We describe briefly several methods supporting the integration of genomic information into the taxonomy and systematics of prokaryotes.

The Average Nucleotide Identity (ANI) (Konstantinidis and Tiedje 2005) measures genetic distance between whole genomes using the conserved reciprocal BLAST best matches. This is derived from genome-scale comparison of short regions (e.g., 10,000 bp) but is not based on genome-scale alignments of the full-length chromosomes. For ANI, 95–96 % sequence identity is generally used for the species delineation (Kim et al. 2014). This number could be somewhat variable depending upon the degree of variation between one species (or cluster of related strains) and the next.

With sufficient ANI data available from related genomes belonging to a single genus, agglomerative clustering algorithms can be used to define species. This allows computation of a variable cutoff for the definition of species within each genus (Logares et al. 2012). A recent publication used ANI values to resolve the *Pseudomonas avellanae* species (Scortichini et al. 2013).

**Fig. 5:**
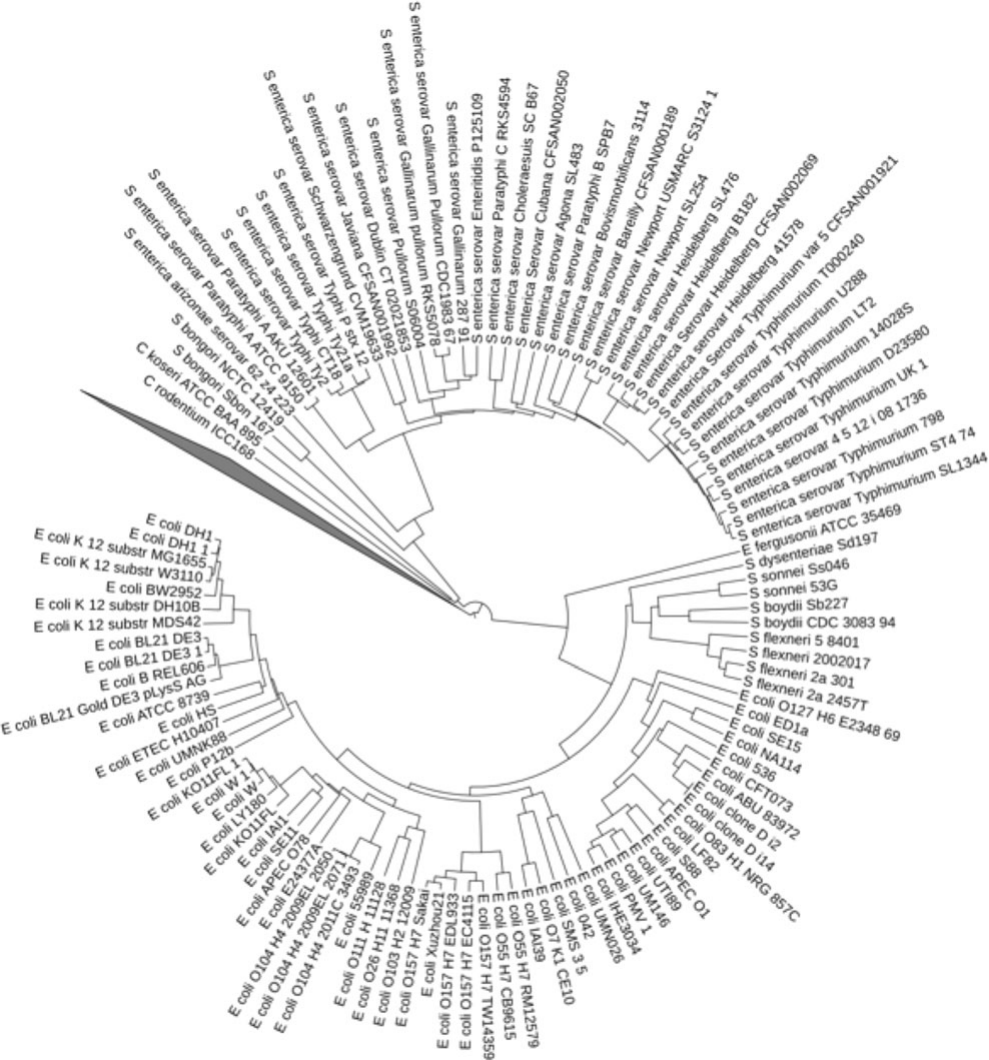
A branching pattern of *E. coli* and *Shigella* on an alignment-free whole proteome phylogeny. Source: data used with permission from whole proteome phylogeny of *E. coli* and *Shigella* by FFP method (Jun et al. 2010)

Another approach within an alignment-free category for whole genome and proteome comparison was proposed by Jun and colleagues (Li et al. 2010). The method used k-mers as features and represented individual whole genomes or proteomes as a Feature Frequency Profile (FFP). In the FFP-based comparison, the most critical issue is determination of the length of the k-mer, which is selected based on three criteria: (1) FFP’s reconstruction capability of whole genome or proteome from FFP, (2) tree convergence, and (3) statistical reliability support. The resulting tree by FFP comparison with an optimal feature length showed that almost all groups were monophyletic at most taxonomic levels (Jun et al. 2010). An example is the branching pattern of *E. coli* and *Shigella* by FFP comparison shown in Fig. 5. Using only the 16S rRNA gene, *E. coli* and *Shigella* grouped with *Escherichia fergusonii*. The figure is a part of FFP-based tree of complete proteomes where *E. fergusonii* separated from the monophyletic group of *E. coli* and *Shigella* (Lukjancenko et al. 2010).

Comparison of genome sequences of closely related bacterial strains has ignited a discussion on the definition of a bacterial species. Phylogenetic trees can be used as a way to visualize and describe genome relatedness and are a starting point for discussion of species boundaries. Trees prepared using different subsets of genes can offer different views on relationships. This has led to the creation of novel concepts of core (genes shared by all or most genomes) and pan (union of genes from all genomes) genome sets (Tettelin et al. 2005). This promises to provide considerable information and insights about species’ relatedness and evolution

One commonly used method of gene tree construction includes the simultaneous examination of several marker genes called MultiLocus Sequence Typing (MLST) (Maiden et al. 1998) or multilocus sequence analysis (Naser et al. 2005). A MSLT pan-genome comparison of *E. coli* and *Shigella,* constructed from the housekeeping genes shows better resolution than trees based on the 16S rRNA gene. It resulted in only 6 % of the pan-genome shared by all genomes (core genome) revealing far greater diversity than expected (Lukjancenko et al. 2010). This is consistent with attempts to use pan-genomics for describing taxonomic and functional diversity. Similar results have been found for *Salmonella*, where several different sets of genes were compared, for calculating MLST (Leekitcharoenphon et al. 2012a, b). Recently, MSLT has been expanded to cover ribosomal protein genes (Jolley and Maiden 2014).

One way to quantify the variation within a group of genomes (from a single species or genus) is to compare the size of their conserved core genome to the size of the combined pan-genome. Since this comparison depends on alignment of protein-coding genes, it is best to standardize the gene identification step for the analyzed genome set. The analysis is surprisingly insensitive to the cutoff parameters for conservation.

**Fig. 6:**
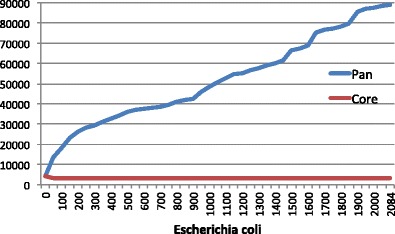
Core and pan-genome of 2085 *E. coli* genomes. Core gene families defined as those families with at least one member in at least of 95 % of genomes

In an effort to tease out biological knowledge, core and pan-genome sizes have been determined for numerous species (Huang et al. 2014). These assessments, however, are dependent on the number of genomes available for analysis. A species that demonstrates the effect of dataset size is *E. coli*. In 2012, with 186 genomes, the number of core gene families was ~3000 gene families (Kaas et al. 2012). In January 2015, the number of *E. coli* genomes had grown to 2085 and a core and pan-genome plot shows that the pan-genome continues to grow, even after more than 2000 genomes (Fig. 6). With the exception of an initial exponential phase, the increase of the pan-genome somewhat linearly correlates to the number of genomes added, while the core genome of 3188 gene families has not changed much since 2012. In this analysis, the *E. coli* pan-genome size is about 90,000 unique gene families. Roughly a third of these genes are singletons—that is, they occur in only one genome. A large percentage of these genomes are draft assemblies, and it is likely that gene fragments and gene calling errors led to an over prediction of unique genes. Even taking this into consideration, there likely are more than 60,000 different *E. coli* gene families, which is an impressive number for a single bacterial species.

### Taxonomic enigmas can be resolved by comparative genomics

Bacterial taxonomy will never reach a fixed state, and the availability of genome sequences has increased the need for considerable reshuffling of groups. The grouping of bacteria based on 16S sequences, DDH and biochemical tests sometimes results in combinations or divisions that are not supported by their genome content. As a result, species, genera, and complete families are being shifted and reordered, in an ongoing process.

Although genome-scale comparisons have proven valuable, genome-based comparison is not yet integrated into the practice of bacterial taxonomy, which by nature is a conservative discipline. The key role that 16S rRNA sequences have played so far causes understandable reluctance to replace it with other measures. This classical taxonomic method is consistent with genome content, in many cases, even though the grouping is sometimes counter-intuitive. The division in the bacterial world between Gram-positive and Gram-negative organisms is deep, and one would expect a corresponding split in the taxonomic relationship of these organisms. There is an odd inclusion of a group of Gram-negative organisms within the Gram-positive *Firmicutes* phylum.

Taxonomic categories above the rank of class are not covered by the Rules of the Bacteriological Code, but the *Firmicutes* phylum is generally accepted, with most of its members belonging to the two large and diverse classes of *Bacilli* and *Clostridia*. The class of *Negativicutes*, whose members all have two membranes and stain Gram-negative, is also placed within this phylum (it is the only member of the order *Selemonadales*). The genus *Veillonella* belongs to these *Negativicutes*, and these bacteria are by no means “rare”: the genus is common in the oral and intestinal microbiota of humans and other animals; in some people, *Veillonella* is the most abundant bacteria on their teeth. Knowing that the *Firmicute* classes are very diverse and in need of pruning, for which 16S sequences are not always a reliable indicator (Ludwig et al. 2009), the genome sequences of *Negativicutes* were tested and their position within the *Firmicutes* was confirmed.

Complete genome sequence comparisons of representatives from several phyla showed the *Negativicutes* were somewhat similar to *Clostridia* (though quite distant) but even more distant to the Gram-negative *Proteobacteria* (Vesth et al. 2013). The genome comparison also confirmed that the *Negativicutes* class was correctly split into two families, *Acidaminococcaceae* and *Veillonellaceaea*. The genes required are to produce an outer membrane resembling those of *Proteobacteria*, but other than that, these species are true *Firmicutes*. These counter-intuitive discrepancies between taxonomic placement and phenotype have been informed by the abundance of sequencing data. An urgent future task is the development new tools based on genome sequence analysis for future taxonomy classification (Chun and Rainey 2014; Ozen et al. 2012).

## New output from metagenomics

Microbial populations occupying unique niches occur in every ecosystem on earth based on their specific metabolic properties. Humans carry ten times more bacterial cells than human cells (Weinstock 2012). Microbes also conduct important roles for biofuels, biocatalysts, and environmental remediation (Desai et al. 2010). Isolate-based genome studies in microbial genomics are limited by the small number of microbes that can be cultured (Rappe and Giovannoni 2003). Faster and cheaper sequencing technologies combined with construction of sequencing libraries removed the barriers of the genomic study of uncultured microorganisms directly from environmental samples; metagenomics is the direct sequencing of the entire DNA isolated from an environment. .

The early stages of environmental “metagenomic” studies targeted 16S rRNA genes to obtain better picture of the species composing the community (DeLong and Pace 2001). The first well-analyzed WGS sequencing metagenomic study of microbial genomes from the environment focused on acid mine drainage (AMD) microbial biofilm by Jill Banfield and colleagues (Tyson et al. 2004). Initially, the sequence data for the more restricted AMD system revealed two major and three to four minor bacterial and archaeal species, although three to four additional species have since been added. Another early WGS metagenomic study reported unexpected community complexity and sequence diversity from the Sargasso Sea surface waters (Venter et al. 2004). During the last 10 years, many big projects and consortia have been launched for sequencing of metagenomes, such as the TerraGenome project (Vogel et al. 2009) for soil. For the human microbiome, the MetaHit project in Europe (Li et al. 2014; Nielsen et al. 2014; Qin et al. 2010), and the Human Microbiome Project (HMP) in the US were established (Huttenhower et al. 2012; Methe et al. 2012). Using many human sampling locations, the HMP sequenced almost 3000 bacteria isolates that were used as the reference genomes for shotgun metagenomics analysis. Using both 16S rRNA sequences and WGS metagenomic sequencing approaches, HMP exploratory sequencing studies of the human microbiome revealed that even healthy individuals differ remarkably in the microbes that occupy habitats such as the gut, skin, and vagina.

In Europe, the MetaHit project focused mainly on human gut microbiome using metagenomic WGS sequencing on DNA extracted from stool samples. From the cohort of 124 European individuals, the first human gut microbial gene catalog was established as 3.3 million non-redundant genes (Qin et al. 2010). The non-redundant gene catalog was recently revised by adding more samples from additional Europeans (Karlsson et al. 2014; Li et al. 2014). The non-redundant gene catalog was used to identify uncultured bacteria in stool samples using co-occurrence information of any genes predicted in the metagenomic clusters. Karlsson et al. and Li et al. showed an improvement in type 2 diabetes prediction by adding metagenomic clusters, (Karlsson et al. 2013; Qin et al. 2012, 2014) indicating the importance of uncultured bacteria in disease pathogenesis. Recently, a rigorous method based on the co-occurrence of gene clusters among metagenome samples was developed which can be used to identify new “metagenomic species” from complex metagenomic samples (Nielsen et al. 2014).

The first and crucial step in a metagenomics study is the collection and processing of the environmental sample. Natural DNA-containing samples such as water, soil, cells, tissue can be collected and filtered. Filtering size should be chosen precisely to get correct target sequences from the environmental sample after removing large cells or debris. In the early metagenomic studies, Sanger shotgun (Sanger and Coulson 1975) sequencing was used for metagenomics (e.g., Venter et al. 2004). Metagenomic shotgun sequencing has shifted to NGS technology with its ability to sequence thousands of organisms in parallel (Caporaso et al. 2012). The substantial improvements in Illumina throughput and read length have helped it dominate metagenomics studies and have promoted substantial increases in the number of metagenomic studies. As of October 2014, the GOLD (Pagani et al. 2012) contains 544 metagenomics studies associated with 6726 metagenome samples and MG-RAST system holds 150,039 metagenomic samples, of which 20,415 are publically available. Third-generation sequencing can create nearly complete genome assemblies of individual microbes directly from environmental samples without the need for cultivation methods (Blainey 2013).

Third-generation sequencing, with its thousands or millions of concurrent sequences, will likely represent a substantial cost reduction over NGS. However, to date, single-molecule reads contain a high fraction of insertions and deletions (indels), although these appear to be stochastic. Third-generation sequencing is an emerging technology, and currently the throughput is quite low. For example, a run can yield a few million bp of DNA, which for a bacterial genome is in the range of 1x coverage or less and this low-level of coverage presents serious challenges for bioinformatics (Quail et al. 2012; Reffaee et al. 2014). It is not clear if the “high error rate” of third-generation sequencing is just a sampling coverage issue—that is, it is possible that the observed indels are “real,” reflecting the variance of individual molecules. When averaged with deeper coverage, agreement with the “consensus sequence” might be achieved. The long reads of third-generation sequencing (average read lengths of ~5000 bp for PacBio, ~10,000 bp for Oxford Nanopore) hold promise for finishing genomes, and for analysis of metagenomic data, which can contain more than 10,000 divergent species with different coverage depth for each species, which makes it harder to analyze the data. Computational challenges rise from simple sample processing, to assembly, binning, and identification of species; further challenges are annotation of genes and of course assignment of function.

The study of metagenomic samples reveal that an organism’s environment is correlated with GC content, genome size, horizontal gene transfer (HGT), optimum growth temperature, and the presence or absence of DnaE2 (Musto et al. 2006; Popa et al. 2011; Raes et al. 2007; Wu et al. 2014). Microbes that inhabit the soil tend to have a higher GC content and larger genome size than their aquatic counterparts. The GC content of bacterial genomes has been shown to be positively correlated to optimal growth temperature and is a major barrier to HGT. Researchers looking for the mechanism involved in land colonization have found DnaE2 in 55–68 % of soil bacteria compared with 11–21 % in bacteria in water (Wu et al. 2014). The correlations between all of these factors and phylogenetic analysis have led them to further speculate that the duplication of *dnaE1* to *dnaE2* followed by an increase in GC where major steps leading to terrestrial bacteria.

## Applications of whole-genome sequencing data

### Transcription unit architecture

Although classical operon structure still seems to predominate, a variety of structures are being found (Conway et al. 2014). Technology is also rapidly changing such that reasonably comprehensive genome-scale transcription unit architectures have been elucidated. The first one for *E. coli*, generated by Palsson and colleagues (Cho et al. 2009) used a variety of techniques including extensive microarray analysis, ChIP-chip for promoter site placement, 5′ RACE for transcription start site (TSS) determination, and shotgun proteomics. This revealed 4661 transcription units (TUs), which was a huge increase over previous determinations, with an average of 1.7 promoters per operon. Unfortunately, this was fairly labor intensive and was not expanded to any other genomes. This data can now be used to create a comprehensive Genetic Regulatory Network (GRN) since the transcription factor-binding sites (TFBS) have recently been determined for virtually all the transcription factors in *E. coli* (Ishihama et al. 2014; Shimada et al. 2014).

A reasonably comprehensive GRN was determined for the archaeal genome *Halobacterium salinarum* NRC-1 (Bonneau et al. 2007). Manual curation of many of the genes was used to catalog 128 transcription factors; analysis of 266 microarrays was then used to create and test software for assembling the GRN. This is a fairly labor-intensive procedure that will not scale very well with increased rate of genome sequencing, although the software may be able to be modified for other studies.

### Genome-scale metabolic modeling with an profusion of sequencing data

Metabolism is the key machinery of livings for cellular operations that are common across different species. With genome sequences, some species-specific metabolic reactions and pathways can be clearly identified (Francke et al. 2005). Based on this concept, the relationship between genotypes and phenotypes by species-specific metabolic network reconstructions at the genome level have emerged and widely apply as Genome-scale Metabolic model (GEM) with constraint-based formulations (Thiele and Palsson 2010). Applying this framework and its derivatives, several studies in microbial evolution, metabolic engineering, biomedical applications, etc. have been highly successful (Bordbar et al. 2014; Monk and Palsson 2014). The first complete genome sequence of the prokaryotic model organism, *E. coli* strain K-12 was publicly released in 1997 (Blattner et al. 1997); 3 years later, the first GEM of *E. coli* (Edwards and Palsson 2000a, b, c) was published and showed promising capabilities to precisely predict cellular behaviors (Edwards and Palsson 2000a, b, c) on the basis of flux balance analysis (FBA) (Orth et al. 2010). This has enabled several developments on large-scale network analysis (McCloskey et al. 2013) that can have several applications (Bordbar et al. 2014). Based on the framework above, several predictive GEMs of prokaryotes (>85 GEMs for Bacteria and >6 GEMs for Achaea) were built and are widely used (Feist et al. 2009). For some organisms, many of the strains were sequenced to identify the fraction of gene contents that can imply the specific phenotype of each strain. Recently, the genome sequences of 55 *E. coli* strains were systematically analyzed for their pan- and core metabolic capability through GEM characterization (Monk et al. 2013). This study demonstrated the capability of GEM in order to predict auxotrophies of different *E. coli* strains that can infer the pathogenicity derived from the mutations.

GEMs reconstruction is not a trivial process and a way to accelerate and automate this process is needed with the profusion of genome sequences. The two well-known comprehensive databases using large-scale metabolic reconstructions, MetaCyc and BioCyc, experienced significant growth in the number of sequenced genomes and their metabolic diversity. In 1999, MetaCyc was described as a manually curated metabolic-pathway database that contained 296 pathways and 3779 metabolic reactions. The numbers increased to 977 and 6483, respectively, in 8 years (Caspi et al. 2008; Karp et al. 1999). A mere 6 years later, MetaCyc contained 2151 metabolic pathways and 11,800 reactions (Caspi et al. 2014).

MetaCyc and BioCyc provide the basis for developing pathway genome databases (PGDBs) and metabolic models for non-model organisms using the Pathway Tools software (Karp et al. 2010). The software includes all necessary components to (1) automatically generate a PGDB for the organism from annotations of the sequenced genome, (2) to query, visualize, analyze, and edit the database, (3) to develop and refine a metabolic-flux model using the PGDB and then to use it for predictions by FBA. Advances in the Pathway Tools software (Latendresse 2014; Latendresse et al. 2012) and the increased number of sequenced genomes dramatically increased the BioCyc collection of automatically generated PGDBs, from only six microorganisms in 1999 to 3563 in the latest version of BioCyc (Caspi et al. 2014). The PGDBs can be further used to generate and solve metabolic-flux models of the microorganisms and to compare their metabolic characteristics in terms of pathways, reactions, enzymes and metabolites.

Another effort for high-throughput generation and analysis of GEMs has been developed under a Web-based environment called Model SEED (Henry et al. 2010). The reconstruction process of the SEED system relies on the annotation from RAST (Aziz et al. 2008) then additional intracellular, transport, and biomass-associated reactions through the proposed auto-completion process (Henry et al. 2010) to make the GEM ready for FBA simulation. Using the SEED pipeline, 130 bacterial GEMs have been built and their quality validated against available gene lethality and Biolog data. Now more than 230 GEMs have been generated and made publicly available through Model SEED (2014).

There are other software platforms that are tailor made for semi-automated GEMs reconstruction like the Raven toolbox (Agren et al. 2013) which has a Web-based version (Garcia-Albornoz et al. 2014) and SuBliMinal toolbox (Swainston et al. 2011) which can be used for high-throughput GEM reconstruction. All mentioned platforms and software were compared in the recent review (Hamilton and Reed 2014) that can be used in practice and further improvements. In addition, researchers now can easily perform GEM reconstruction and FBA simulation through open software on the Website provide by the Department of Energy Systems Biology Knowledgebase (KBase) at www.kbase.us

### Infectious disease epidemiology

As the world watches for the next flu pandemic, sudden appearance of deadly *E. coli*, Ebola outbreak, or even bioterrorism, the capabilities of biosurveillance and bioforensics are becoming increasingly important parts of life. Genome sequencing is an important driver in the development of databases, tools, and algorithms being developed to detect and ward off the threats (Francis et al. 2013; Schriml et al. 2007). Rapid and targeted identification of pathogens is now seen as an important component of an effective response during an epidemic (Koehler et al. 2014).

WGS holds the promise to revolutionize surveillance and diagnostics of infectious diseases due to its high resolution. It may be used across many areas such as monitoring food, environment, clinical, veterinary, wildlife, etc.*,* for all known pathogens, i.e.*,* viruses, bacteria, fungi, parasites, etc. A major obstacle is how to create a robust and simple to use system that will allow its adaptation within the relevant labs. A goal would be to establish a Web-based system, allowing users to upload sequence and meta data for several isolates in one batch upload, and have several analysis made on each isolate: assembly, species typing, MLST typing (for bacteria), resistance gene finding, virulence prediction, and gene finding. Furthermore the system should allow single nucleotide polymorphism (SNP) based comparison of the uploaded isolates with all previously uploaded isolates.

A limited version of such as this system has been running since 2012 (CGE 2014). The beta version is expected to be operational in 2015. So far, more than 72,000 isolates have been analyzed. This has demonstrated that online analysis of WGS information is possible. This means it should be possible to create a unified portal so that all area and pathogen data can be compared, enabling us to trace back all infections.

The Center for Genomic Epidemiology (CGE) has, over the last 4 years, worked on developing a system for surveillance and diagnostics of infectious diseases. The basic aims have been to develop methods to find out what is in a sample (typing), how pathogenic it is, and what the antibiotic resistances profile is (phenotyping). For epidemiological tracing it is furthermore necessary to know how a given isolate is evolutionarily related to other isolates. An over overall description of the aims can be found in Aarestrup et al. (2012), and all the methods developed are available online (CGE 2014).

The first tool developed at CGE was a method for MLST of bacteria using the raw reads (or assembled genomes) as input (Larsen et al. 2012). As for other MLST methods, the user must know the species for the method to use the correct MLST scheme. A number of methods to deduce the species from the raw sequences were therefore developed based on the16S rRNA gene, k-mers, and ribosomal and core genes (Larsen et al. 2014). It was found that a k-mer-based method was very fast and reliable for species identification.

Once a pathogen is diagnosed, it is important to know how it can be treated—what treatments are likely to work, and which treatments are likely to be ineffective or harmful. A method has been developed for identification of acquired antimicrobial resistance genes (Zankari et al. 2012). A major effort was put into compiling a human-curated database based both on public databases and scientific papers. Concerns have been raised because an assigned genotype may not always correspond to a phenotype. For example, mutations outside a gene may affect the expression of the gene product. A study was therefore conducted to compare geno- and phenotypes. It was found that genotyping using WGS is a realistic alternative to surveillance based on phenotypic antimicrobial susceptibility testing (Zankari et al. 2013), and a surprisingly high concordance (99.74 %) was found between phenotypic and predicted antimicrobial susceptibility. This is promising, but the study was conducted in a population with relatively low levels of resistance and lower levels of concordance may be found in other populations.

The methods described above are all based on alignment to a database of genes with known (pheno-) types. Andreatta et al. took a radically different approach and sorted genomes of Gamma-Proteobacteria into pathogenic or non-pathogenic, and looked for gene families that were statistically associated with either pathogenic or non-pathogenic bacteria (Andreatta et al. 2010). This is perhaps the first example of using machine learning techniques to determine the phenotype from WGS. The method was later extended to work for all species of bacteria using raw sequencing data as input (Cosentino et al. 2013).

Similar methods can also be used on the single protein level. Jessen et al. developed a method for finding sites associated with biological activity. Based on sorting the sequences, the measured activity associated with each sequence was then statistically investigated if certain amino acids at certain positions we associated with biological activity (Jessen et al. 2013).

Much attention has recently been given to the possibility of diagnosing diseases based on metagenomic samples. This is faster and simpler than having to isolate the bacteria. Hasman et al. have shown that metagenomic samples (in this case urine) could be used to diagnose a pathogen without prior knowledge about which species. It was found that WGS improved the identification of the cultivated bacteria, and an almost complete agreement was observed between phenotypic and predicted antimicrobial susceptibilities (Hasman et al. 2014). For this project, ChainMapper was developed to map all reads against all fully sequenced bacteria and viruses, as well as resistance genes and genomes from the MetaHIT project. This method has since been updated and re-implemented and is available via a method called MGmapper.

Making phylogenetic trees based on SNPs is the emerging standard for detailed study of evolutionary relationships between isolates in an outbreak. Leekitcharoenphon et al. recently developed the first Web-based server for SNP tree analysis (Leekitcharoenphon et al. 2012a, b). In SNP tree analysis the details of the method such as how SNPs are called and filtered are very important to the reliability of the result. Work to evaluate and refine phylogeny methods have resulted in the NDtree and CSIphylogeny methods, which both were shown to be more accurate than the original SNPtree method (Kaas et al. 2014; Leekitcharoenphon et al. 2014).

## Bioinformatics and computational infrastructure

Computers are playing an increasing role in sequencing and analysis. Biological problems are no longer confined to the study of one gene, one genome, one sequence per genome, or even a small number of related genomes. Like physics, biology has become a big-data science, but with more complex data types from a variety of sources. The exponential growth has been quite sudden, and it is easy to under estimate the magnitude of the problem. Many public funding agencies are demanding detailed plans for how data will be stored, archived, and accessed. This was easy for small sets of data, but is more difficult when a study includes thousands of sequenced genomes, with thousands of phenotypes or growth conditions and the integration of multiple “omics” data. Grant review committees are starting to ask about computational capabilities necessary to deal with the large amount of data being generated.

It is easy to think that buying a new, faster computer will help manage the ever-growing number of available genomes. Computers are getting faster and can store more data—this has been going on for many years, and this is related to Moore’s law; in 1965, Gordon Moore published an article estimating that the number of transistors on an integrated circuit was doubling every 2 years. This trend has continued for five decades now, although the estimate for doubling time has been revised to about 1.5 years (18 months).

In the past 20 years, computing capabilities have grown about 10,000-fold—that is, computers can now store and process 10,000 times as much information than they could when the first bacterial genome was sequenced. The sequencing technology, however, has improved much faster—there are roughly a hundred thousand bacterial genomes sequenced now, compared with two genomes in 1995.

Further complicating matters is that many methods use pairwise comparisons of genomes, which squares with the size of the database. To compare 2000 genomes will take four million times as much computational power as was needed to compare the first two genomes.

As was noted in Fig. 4, GenBank has grown more than 250,000-fold from when it was first formed, in 1982. But GenBank no longer contains all the sequence data. The Sequence Read Archive was formed in 2007 as a depository for short sequence reads, which continues to grow and currently it is about 2000 times as large as GenBank. In addition, thousands of metagenomic data samples are not included in the counts of sequences in GenBank and the SRA. In addition to genome sequences, data analysis often includes transcriptomics data (“RNAseq”), as well as proteomics, metabolomics, etc. Data is being generated at far greater speeds than computers are improving, presenting challenges to biological researchers. As noted above, biological data is heterogeneous, requiring the development of data models that and many experimental biologists are carefully structured and linked for rapid retrieval of related information. For some applications, such as epidemiology monitoring or biosurveillance, timeliness is critical—results that cannot be provided quickly may be useless. New approaches to assessing the quality of data will need to be explored. With larger and richer datasets, privacy concerns are increased—an important consideration for researchers studying human microbiota.

If processing speed was the only impediment, it could be argued that moving to high-performance supercomputers would solve the problem. Experts are working on exascale computing—the processing of one exaFLOPS, or a billion billion calculations in 1 s. If the number of sequences grows at its current rate or faster, soon even high-throughput computing on the fastest computers in the world might not be enough to keep up and it is only one aspect of the shortfall. There are problems with the logistics of storing and computing this data and—very real problems of how to visualize the data. For example, with today’s 20,000 bacterial genomes and approximately 5,000 genes per genome, an all vs all protein comparison would take 4 months at the rate of a billion billion comparisons per second. Charts, plots, Venn diagrams, and other static images designed for presentation on paper, provide a visual means of comparing a small number of proteins, growth conditions, or genomes. These traditional methods do not easily lend themselves to a comparison of 2000 *E. coli* genomes. Supercomputers can help, but fundamentally different approaches need to be taken into consideration, as we go from terabytes of data to petabytes and soon to exabytes.

There have been significant advances in computing technologies over the past decade. Data storage systems have increased in capacity and decreased in cost by orders of magnitude as the technology has transitioned from magnetic tapes and disks to distributed cloud storage spanning hundreds or thousands of physical devices. Dramatically reduced storage costs have facilitated and encouraged the collection of massive amounts of data across many scientific disciplines, including those in the life sciences. In genomics, decreases in DNA sequencing costs closely tracked decreases in data storage costs until 2008, when the advent of second-generation sequencing significantly accelerated decreases in sequencing costs; note that in Fig. 4, Moore’s law appears to be a flat line at the bottom of the graph, compared with the SRA. Further decreases in sequencing costs are being realized as third-generation sequencing platforms come online. With reduced costs have come increases in the volume of archived sequence data and concomitant efforts to develop scalable data models that provide fast, flexible access. NoSQL (Not Only SQL) databases, including document databases, such as MongoDB, big table databases, such as Accumulo, and graph databases, such as Neo4j, are increasingly used to organize metadata associated with biological sequences, facilitating quick access to related data and construction of biological networks, including metabolic, regulatory, transcriptional, and signaling.

Computing systems have continued to increase in power over the past decade as the number of processing cores in the largest machines has expanded from thousands to millions. A corresponding performance improvement of four orders of magnitude has resulted, with the number of operations per second increasing from the trillion (10^12^) to the quadrillion (10^15^) range. To take advantage of this opportunity for massive parallelism, bioinformatics application programs are being reorganized with multiple threads, code sections that can be executed concurrently, particularly in the areas of sequence analysis, phylogenetics, and functional genomics. This refactoring task is made more difficult when the target high-performance computing platforms are hybrid architectures that combine conventional processors with graphics processors and employ both shared and distributed memories with multiple cache levels.

In the coming decade, efforts to improve the capacity, density, reliability, stability, and speed of storage technologies will continue. Potential new technologies include holographic storage (Timucin and Downie 2000), which uses light to read and write data stored in three-dimensional media, the use of DNA as a storage medium (Church et al. 2012), and atomic-scale magnetic memory (Loth et al. 2012). More powerful computing systems will be built with billions (10^9^) of processing elements and unprecedented levels of concurrency. Research in quantum computing, with the promise of rapid solutions to complex search and optimization problems, will continue. When quantum computers are realized, they will execute quantum programs already developed for comparative genomics tasks, including the identification of mutations in biological sequences (Gueltas et al. 2014).

## Where are we going? Future directions

Cheap, reliable, and fast DNA/RNA sequencing can be used to completely transform infectious disease epidemiology and biosurveillance in the near future. Sequencing will eventually replace most of the other diagnostic tests and detection mechanisms, and therefore, fast and robust bioinformatic analysis tools will be needed to reliably handle this data deluge. These tools will need to provide physicians with fast and accurate diagnoses at the push of a button, and epidemiologists and biosurveillance experts with timely data for tracking outbreaks in geospacial real time without the use of supercomputers.

Three recent publications discuss various aspects of this future and give excellent recent examples of successful implementation WGS for outbreak monitoring, control, and forensics.

The first (Köser et al. 2012) is a much more practical discussion on how to implement the routine use of WGS for diagnostics, biosurveillance, and public health benefits. Since this is from the Sanger Center and their collaborators who have actually published on successful applications of WGS (Harris et al. 2013) in an Methicillin-resistant *Staphylococcus aureus (*MRSA) outbreak monitoring and control in a hospital, as well as its distribution among humans and their animal companions, it is really a blueprint for implementation at the regional and national level.

The second (Croucher et al. 2013) discusses both the theoretical and practical implementation of WGS. The emphasis is on the different requirements of WGS for either local or global questions. In local outbreaks timely high-resolution data resulting in complete genomes would be ideal but is still impractical in the near future due to the cost. However, nearly complete draft genomes mapped to complete reference genomes should suffice. In addition, they discuss the need to differentiate between true variants and false positive and false negative results due to systematic technology errors, bioinformatics errors such as incorrect mapping, and problems caused by recombination.

The third (Kao et al. 2014) is mostly a theoretical discussion of how to use the data and is particularly centered around how to model the data for different applications. They discuss different modes of transmission versus different problems with sampling schemes and the problems of types and rates of exchange between humans and animal pathogen reservoirs.

Although not commercially available yet, the Oxford Nanopore MinIon sequencer is a step in that direction, since it is not much bigger than thumb drive and plugs into the USB port of a laptop computer. This allows one to imagine the following scenario. A young child is brought into a pediatrician’s office with a severe upper respiratory tract infection. The nurse collects some sputum from the child’s nose and places it into a machine that does a simple sample preparation. Thirty minutes later, the nurse collects the processed sample and puts it into a MinIon like sequencer, which is plugged into a laptop and pushes the start button. In another hour, she gets a diagnosis from the laptop and prescribes the appropriate antibiotic for a bacterial infection or some other treatment if it is a virus. A MinIon run gets usable reads in as little as 10 min. Although this is longer and more expensive than the current tests for “strep throat” or the “flu,” it would give definitive diagnoses for virtually all other pathogens, especially for difficult to diagnose ones like *Bordetella pertussis,* whose current test takes 3–4 weeks.

Finally, there is recent evidence that gut microbiota effects eating behavior, weight, and moods (Heijtza et al. 2011; Kocelak et al. 2013). This subject will be of great interest in the food industry, and high-throughput microbial genome sequencing will be an important tool in these studies. As we gain knowledge about how our gut microbes affect our behavior via the vagus nerve, the microbial hormone, and neurotransmitter production, or via the cannabinoid and opioid receptors, this information can be used to not only to produce probiotics that can increase the quality of our health, but also could influence our eating behavior so as to crave certain foods that a production company would like to sell us.
